# Telovelar approach for microsurgical resection of fourth ventricular subependymoma arising from rhomboid fossa: operative video and technical nuances

**DOI:** 10.3171/2019.10.FocusVid.19452

**Published:** 2019-10-01

**Authors:** James K. Liu, Vincent N. Dodson

**Affiliations:** Department of Neurological Surgery, Center for Cerebrovascular and Skull Base Surgery, Neurological Institute of New Jersey, Rutgers University, New Jersey Medical School, RWJ Barnabas Health, Newark and Livingston, New Jersey

**Keywords:** tela choroidea, velum, telovelar, rhomboid fossa, subependymoma, video

## Abstract

Fourth ventricular tumors have traditionally been removed via transvermian approaches, which can result in potential dysequilibrium and mutism. The telovelar approach is an excellent alternative to widely expose fourth ventricular tumors without transgressing the cerebellar vermis. This is achieved by opening the cerebellomedullary fissure and incising the tela choroidea and inferior medullary velum, which form the lower half of the roof of the fourth ventricle. In this operative video manuscript, the authors demonstrate microsurgical resection of a fourth ventricular subependymoma arising from the rhomboid fossa via the telovelar approach. The key technical nuance in this video is to demonstrate a gentle and safe technique to identify a dissectable plane to peel the tumor off of the rhomboid fossa using a microspreading technique with fine micro-bayonetted forceps. A gross-total resection was achieved, and the patient was neurologically intact.

The video can be found here: https://youtu.be/ZEHHbUGb9zk.

## Transcript

### 0:20–0:32 Title

This is Dr. James Liu, and I’ll be demonstrating an operative video on the telovelar approach for microsurgical resection of a fourth ventricular subependymoma arising from the rhomboid fossa.

### 0:33–0:54 Patient history

The patient is a 35-year-old female who presented with a known fourth ventricular tumor that was initially incidentally detected back in 2013 during a workup for hyperprolactinemia. The mildly elevated prolactin was attributed to the effects of antipsychotic medications and imaging was negative for a pituitary tumor.

### 0:55–1:35 Preoperative imaging

The tumor appeared hyperintense on T2 and FLAIR images. There was also compression along the floor of the fourth ventricle, displacing the brainstem anteriorly. The lesion was isointense on T1-weighted images and did not enhance after gadolinium, suggestive of a diagnosis of subependymoma. Although the lesion was obstructing the fourth ventricle, there was no evidence of hydrocephalus. Comparison to prior imaging studies demonstrated progressive growth over the course of 3 years. Given the patient’s young age, tumor progression, and high risk of obstructive hydrocephalus, the decision was made to surgically intervene.

### 1:35–2:17 Introduction to the telovelar approach

We chose a telovelar approach to access the fourth ventricle. This is achieved by mobilizing the cerebellar tonsils laterally and identifying the tela choroidea and inferior medullary velum, which form the lower half of the roof of the fourth ventricle. The arachnoid here is opened sharply as seen on the yellow dotted line to offer wide access to the fourth ventricle without any violation of the cerebellar vermis. The key technical nuance in this video is to demonstrate a gentle and safe technique to identify a dissectable plane to peel the tumor off of the rhomboid fossa using fine micro-bayoneted forceps.

### 2:17–2:40 Patient positioning

The patient was placed in the prone Concorde position with the head in three-pin fixation in a Mayfield head holder. A standard midline craniocervical skin incision was used. We used intraoperative navigation and neuromonitoring with EMG of the facial nerve, lower cranial nerves IX–XII, auditory brainstem responses, and somatosensory and motor evoked potentials.

### 2:40–3:13 Suboccipital craniectomy and laminectomy

After incising the skin, midline dissection down to the bone followed by subperiosteal elevation of the soft tissues exposed the subocciput down to the C2 lamina. We then performed a midline suboccipital craniectomy C1 and superior partial C2 laminectomy to obtain an adequate trajectory and viewing angle to the fourth ventricle. The atlantooccipital membrane was then removed. The dura was opened in a Y-shaped fashion and the dural leaflets were tacked up with 4-0 Nurolon sutures.

### 3:14–4:15 Exposure of cerebellomedullary fissure and tela choroidea

Using the intraoperative microscope, we opened the arachnoid sharply over the cisterna magna and identified the cerebellar tonsils and spinal cord. The gray-appearing tumor was protruding from the foramen of Magendie, displacing the tonsillomedullary segments of PICA laterally. The left cerebellomedullary fissure is opened sharply, freeing the arachnoid from PICA. The tela choroidea was thinly stretched over the tumor and incised superiorly towards the choroid plexus.

A plane was developed between the tumor and the spinal cord using gentle spreading of the microforceps. The cerebellomedullary fissure was opened on the right side, separating the arachnoid from the right PICA. Microscissors are used to lyse the adhesions.

### 4:15–7:29 Resection of tumor

Incising the tela choroidea provided excellent exposure of the tumor arising from the fourth ventricle. We began with central debulking of the tumor with an ultrasonic aspirator.

Extracapsular dissection is performed with the micro-bayonetted forceps. The tela choroidea was separated from the left lateral aspect of the tumor. This was also performed on the right side in the right cerebellomedullary fissure. Further debulking of the inferior pole of the tumor was performed with special consideration to protect the rhomboid fossa.

We then turned our attention to the superior pole of the tumor. The arachnoid of the tela choroidea and inferior medullary velum were incised. Care was taken to separate and preserve the small perforators surrounding the tumor. Further debulking of the superior pole of the tumor was performed. We then dissected the tumor carefully away from the choroid plexus.

After the superior pole of the tumor is significantly debulked, the tumor can be elevated to expose the floor of the fourth ventricle. While debulking the tumor, it is essential to ensure protection of the rhomboid fossa by using a cottonoid patty. This serves as a useful marker and preserves the dissection plane. The patty is advanced inferiorly as the tumor is sequentially debulked.

The remaining portion of the tumor was arising from the inferior aspect of the rhomboid fossa. We interrogated the plane to see if the tumor could be separated from the floor of the fourth ventricle. Meticulous and gentle spreading with the microforceps allowed us to develop this plane. EMG as well as motor evoked potentials of both cranial nerves X and XII were performed as we dissected the remainder of the tumor from the vagal and hypoglossal trigones.

The plane of dissection was very favorable, and there was no irritation detected from the neuromonitoring. Therefore, we proceeded to carefully peel the tumor off of the rhomboid fossa. The last point of attachment was divided with sharp dissection. This technique and strategy allowed us to achieve a gross-total resection. The motor evoked potentials remained stable at baseline.

### 7:29–7:46 Closure

Hemostasis was achieved and the dura was closed in a watertight fashion with an AlloDerm patch graft. A cranioplasty was performed with a suboccipital titanium plate. The wound was closed in a standard multilayered fashion.

### 7:46–8:13 Postoperative course

Immediate postoperative imaging shows gross-total resection of the tumor. CSF outflow from the fourth ventricle is completely unobstructed. Follow-up imaging 2.5 years later demonstrates no recurrence of tumor. Postoperatively, the patient was neurologically intact. Final pathology confirmed a WHO grade I subependymoma.

### 8:13–8:33 Conclusion

In summary, the telovelar approach provides excellent access to fourth ventricular tumors without violation of the cerebellar vermis. It is imperative to preserve the rhomboid fossa to avoid neurological injury (Strauss et al., 1997, 1999; Mussi and Rhoton, 2000; Jittapiromsak et al., 2010; Tomasello et al., 2015; Winkler et al., 2016).

## Disclosures

The authors report no conflict of interest concerning the materials or methods used in this study or the findings specified in this publication.

## Supplemental Information

Supplementary Figs. 1 and 2
