# Supplementary material for: Telovelar approach for microsurgical resection of fourth ventricular subependymoma arising from rhomboid fossa: operative video and technical nuances
Source: Neurosurg Focus Video. 2019 Oct 1;1(2):V5. doi: 10.3171/2019.10.FocusVid.19452 (PMC9541721; doi:10.3171/2019.10.FocusVid.19452)
Supplement: Supplementary Figs. 1 and 2 [file SupplementaryFigs1and2_OCtFocusVid19452.pdf]

Supplemental material

Telovelar approach for microsurgical resection of fourth ventricular subependymoma arising from rhomboid fossa: operative video and technical nuances

James K. Liu, MD and Vincent N. Dodson, BS

http://thejns.org/doi/abs/10.3171/2019.10.FocusVid.19452

DISCLAIMER *Neurosurgical Focus: Video* acknowledges that the following section is published verbatim as submitted by the authors and did not go through either the journal's peer-review or editing process.

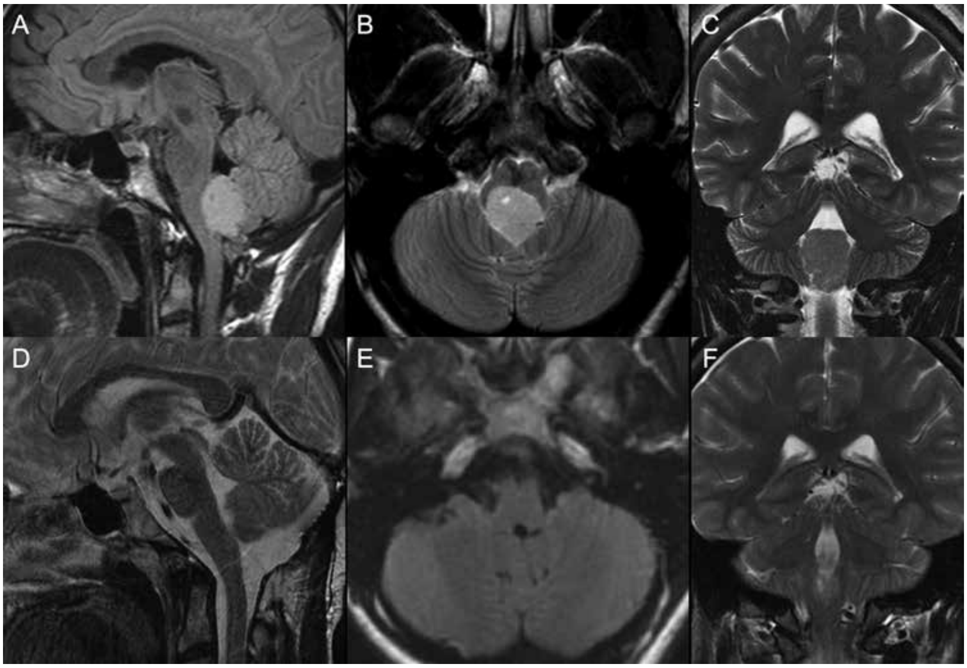

FIGURE 1. Preoperative sagittal T2-FLAIR (A) and axial (B) and coronal (C) T2-weighted MRI demonstrate a hyperintense mass along the floor of the fourth ventricle, compressing the brainstem anteriorly. Postoperative (2.5 years) sagittal T2-weighted (D), axial T2-FLAIR (E), and coronal T2-weighted (F) MRI demonstrate unobstructed CSF outflow from the fourth ventricle and no recurrence of tumor.

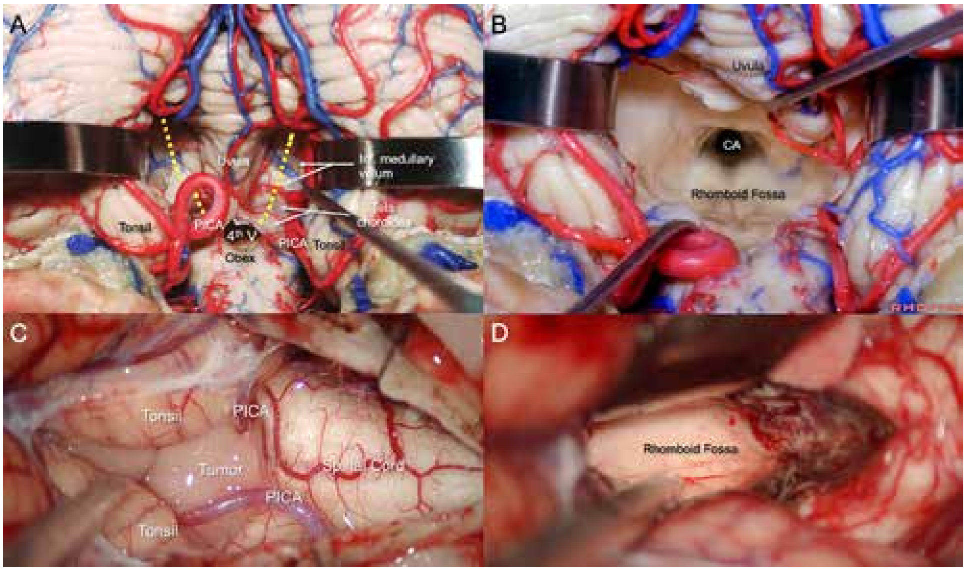

FIGURE 2. Cadaveric dissection demonstrating that the incision of the tela choroidea and inferior medullary velum (A, yellow dotted line) provides wide access to the fourth ventricle (B) without violating the cerebellar vermis. Courtesy of The Rhoton Collection. C: Intraoperative image demonstrating the gray-appearing tumor protruding from the foramen of Magendie, displacing the tonsillomedullary segments of PICA laterally. D: Intraoperative image demonstrating resection bed free of any tumor. Gross-total resection was achieved.
